# Body mass index and the risk of ulnar nerve entrapment in individuals without diabetes—a longitudinal cohort study from Sweden

**DOI:** 10.1038/s41366-025-01899-y

**Published:** 2025-09-17

**Authors:** Mattias Rydberg, Lars B. Dahlin, Peter M. Nilsson, Malin Zimmerman

**Affiliations:** 1 Department of Hand Surgery, Lund University, Skåne University Hospital, Malmö, Sweden; 2 Department of Translational Medicine—Hand Surgery, Lund University, Lund, Sweden; 3 Department of Internal Medicine, Clinical Research Unit, Lund University, Malmö, Sweden; 4 Department of Clinical Sciences, Lund University, Skåne University Hospital, Malmö, Sweden; 5 Department of Orthopedics, Helsingborg Hospital, Helsingborg, Sweden

**Keywords:** Risk factors, Obesity, Diseases of the nervous system

## Abstract

**Introduction:**

Ulnar nerve entrapment (UNE) is a common disorder with many associated risk factors. Diabetes mellitus (DM) is an established risk factor, but less is known about metabolic risk factors in individuals without diabetes. Our study aimed to explore the association of body mass index (BMI) with UNE during long-term follow-up.

**Method:**

The population-based cohort study Malmö Diet and Cancer Study (MDCS) and the Swedish Patient Register (NPR) were cross-linked. Between 1991 and 1996, 30,446 subjects were recruited to MDCS and were followed to a diagnosis of UNE, emigration, death, or end of study on December 31, 2020. BMI at study entry was stratified into normal weight (<25), overweight (25–30) and obesity (>30). To omit the effect of DM, individuals with prevalent or incident DM were excluded. To calculate the association between BMI and incident UNE, Cox proportional hazard models adjusted for age, sex, hypertension, smoking, manual work, and alcohol consumption were used.

**Results:**

A total of 23,254 individuals were followed for over 25 years, whereof 192 (0.8%) developed UNE. In the multivariable Cox regression models, BMI was independently associated with UNE (HR 1.07; 95% CI 1.03–1.11, *p* < 0.001). Both overweight (HR 1.55; 95% CI 1.12–2.15, *p* < 0.01) and obesity (HR 2.23; 95% CI 1.40–3.57, *p* = 0.001) were associated with an increased risk compared to individuals with normal weight.

**Conclusion:**

High BMI is associated with the development of UNE in individuals without diabetes, indicating that high BMI is an independent risk factor for the development of nerve entrapment disorders irrespective of hyperglycaemia.

## Introduction

Ulnar nerve entrapment (UNE) is a nerve entrapment disorder, second in prevalence only to carpal tunnel syndrome (CTS). The ulnar nerve passes dorsally to the medial epicondyle in the elbow, where the ligament of Osborne is one location that makes the nerve prone to compression in the cubital tunnel [1]. The prevalence is estimated to be ~1–2% in the general population but is substantially higher in individuals with diabetes mellitus (DM) [2–4]. Symptoms of UNE include numbness and paraesthesia in the little and ring finger, muscle weakness, and, in severe cases, even muscle atrophy of the ulnar-innervated interossei muscles in the hand, with clawing and pain can be a prominent feature of the disorder [5]. The exact aetiology and pathogenesis of UNE are not fully understood and are still being debated; however, there are several proposed risk factors for which the most robust evidence exists for DM [6], smoking [7], and occupations with a heavy workload and vibration exposure [8, 9].

Recent publications have tried to elucidate the effect of hyperlipidaemia and obesity on nerve function and peripheral neuropathy [10–12]. As most previous studies have focused on and included individuals with DM, far less is known about peripheral neuropathy, and particularly UNE, in individuals without DM. Thus, this study aims to explore the association of high body mass index (BMI) and incident UNE in individuals without DM while adjusting for other known risk factors in a large cohort from southern Sweden, including over 30,000 individuals during long-term follow-up.

## Methods

### Study sample and baseline characteristics

Data from the population-based Malmö Diet and Cancer Study (MDCS), a cohort of 30,446 individuals from southern Sweden, was used in the study. Participants, aged 46–73 years, were recruited at baseline between 1991 and 1996, underwent clinical examination and laboratory assessment, and filled in a questionnaire regarding basic screening for cardiovascular risk factors. Originally, the MDCS sought to assess the link between diet and cancer, and the cohort has been previously described in detail in several studies [13]. Upon recruitment, a trained nurse assessed all participants’ height, standing with a fixed stadiometer, and weight, measured to the nearest 0.1 kg with a balance-beam scale. BMI was calculated as kg/m^2^. Blood pressure (BP) was measured in a supine position using a sphygmomanometer, and hypertension was defined as a systolic BP ≥ 140 mm Hg or a diastolic BP ≥ 90 mm Hg. Smoking was self-reported and defined as a current smoker or non-smoker. Consumption of alcohol was also self-reported as total consumption during the last week in grams per day. DM at baseline was defined as fasting whole blood glucose > 6.0 mmol/L at baseline, a self-reported physician’s diagnosis, or the use of antidiabetic medicine. Data on incident and prevalent DM were also obtained using six national and local registers previously described in detail [14]. Also previously described in detail, individuals’ occupations were based on self-reported job titles and classified as manual workers, including farming and non-manual workers [15]. All participants provided informed consent, and the study was approved by the ethical committee at Lund University as well as the Swedish Ethical Review Authority (DNR: LU51-90; 2009-633; 2019-01439).

### Endpoints

From the Swedish patient register (NPR) and cause of death register by the National Board of Health and Welfare (http://socialstyrelsen.se/english), primary endpoint diagnosis, i.e., UNE, was obtained using each participant’s unique 10-digit personal number [16]. Using the International Classification of Diseases (ICD) version 9 or 10, a diagnosis of UNE was retrieved using the ICD 8, 9 and 10 codes: 352.01; 354 C; G562. Only clinical, hospital-based diagnosis codes were available in this study, and surgery codes were not included. Data from primary care and, e.g., occupational therapists were not available. NPR has previously been extensively reviewed and has been shown to have a high case validity, both for neurological and musculoskeletal diagnoses [17–19].

### Statistical analysis

**Fig. 1:**
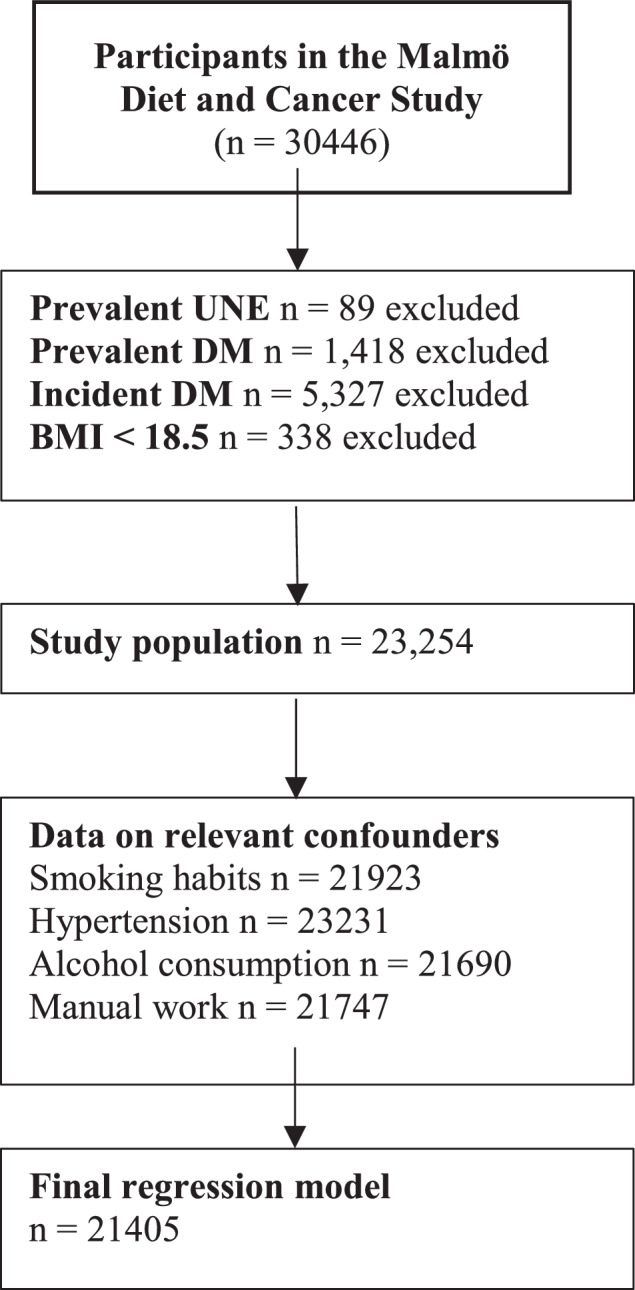
Derivation of the study cohort from the Malmö diet and cancer study. BMI body mass index, DM diabetes mellitus, UNE ulnar nerve entrapment.

Individuals were followed from baseline until either a diagnosis of UNE, emigration, death, or the end of study 2020-12-31, thus creating a time-to-event variable unique for all participants. Individuals with a previous diagnosis of UNE at baseline (*n* = 89) were excluded. BMI was divided into three categories: <25 (normal weight), 25–30 (overweight), and ≥30 (obese) kg/m^2^. All participants with a BMI < 18.5 were excluded since the number was small (*n* = 338) and to exclude the effect of underweight individuals. Furthermore, to exclude the effect of DM and hyperglycaemia on incident UNE, individuals with either prevalent DM (*n* = 1418) or incident DM during follow-up (*n* = 5327) were excluded. (Fig. 1) All baseline data were presented as mean or median for quantitative data and count and proportion nominal data, respectively.

Cox proportional hazard regression models were used to calculate differences in incident UNE between BMI groups and for BMI as a continuous variable. Two models were used; the first model adjusted only for age and sex, and the second model adjusted for age, sex, hypertension, smoking habits, manual work, and alcohol consumption. Hazard ratios (HR) with 95% confidence intervals were expressed with the normal weight group as a reference and for a one-unit increase in BMI, respectively. Furthermore, Kaplan–Meier plots were used to calculate the cumulative incidence of UNE with corresponding log-rank tests to compare groups. The assumption of proportional hazard was assessed by observing the Kaplan–Meier curves and log-log plots, and no violation was found. Finally, two sensitivity analyses were created; the first excluded all participants with a BMI < 20 (*n* = 878), and the second excluded all participants with a follow-up time >20 years (*n* = 16,302), as well as an interaction model between BMI and smoking (BMI*smoking) that was created and analysed.

All statistical analyses were conducted using SPSS for Mac version 27 (SPSS Inc., Chicago, IL, USA). A two-tailed *P* value < 0.05 was considered significant.

## Results

### Baseline characteristics

After excluding individuals with prevalent (*n* = 1418) or incident DM (*n* = 5327), as well as individuals with prevalent UNE at baseline (*n* = 89) and underweight individuals with a BMI < 18.5 (*n* = 338), the total study population consisted of 23,254 individuals. These were followed for a median of 25 years, and 192 (0.8%) individuals developed UNE during the study period. All baseline characteristics can be found in Table 1.

**Table 1:** Participant characteristics at baseline, stratified for BMI subgroup.

Variable	Normal weight *n* = 11,946	Overweight *n* = 8947	Obesity *n* = 2361
Age, years (±SD)	56 ± 7	59.6 ± 8	59 ± 7
BMI (kg/m^2^) (±SD)	22.6 ± 1.6	27.0 ± 1.8	32.7 ± 2.7
Current smoking (n, %)	3663 (32.2)	2024 (24.1)	435 (20.1)
Alcohol (g/day, IQR)	7.5 (13.0)	7.6 (14.2)	5.1 (12.5)
Hypertension (*n*, %)	4282 (35.9)	4338 (48.5)	1445 (61.2)
Manual work (*n*, %)	3572 (31.7)	3231 (38.8)	1036 (48.5)
Incident UNE (*n*, %)	82 (0.7)	80 (0.9)	30 (1.3)

*BMI* body mass index, *IQR* interquartile range, *SD* standard deviation, *UNE* ulnar nerve entrapment.

Normal weight; BMI < 25, overweight; BMI ≥ 25 to <30, obesity BMI ≥ 30 kg/m^2^. Participants with prevalent or incident DM, with a BMI < 18.5 or prevalent UNE, were excluded.

### Survival analysis

**Fig. 2:**
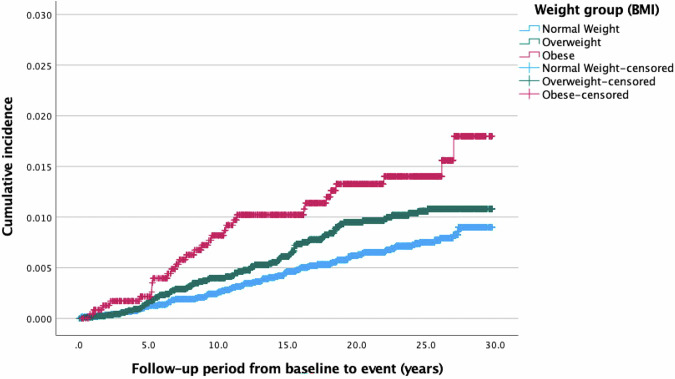
Kaplan–Meier survival plot with cumulative incidence of UNE, based on diagnosis without surgical codes, stratifying for weight group. Log-rank test for difference in survival distributions *p* = 0.002. UNE ulnar nerve entrapment.

In the Kaplan–Meier plots, there was longer UNE-free time in the group with normal weight than in both groups with overweight and obesity (log-rank test *p* < 0.002) (Fig. 2).

In the first crude Cox regression model adjusted for age and sex, both overweight (HR 1.49; 95% CI 1.08–2.06, *p* = 0.02) and obesity (HR 2.07; 95% CI 1.31–3.28, *p* < 0.01) were associated with incident UNE during follow-up. Further adjustments for smoking, manual work, hypertension, and alcohol consumption in the second model did not significantly change the results (Table 2). When analysing the results from BMI as a continuous variable (kg/m^2^) in the Cox regression model, one unit increase in BMI was associated with incident UNE both in the crude model (HR 1.06; 95% CI 1.02–1.10, *p* < 0.01) and the fully adjusted model (HR 1.07 95% CI 1.03–1.11, *p* < 0.001) (Table 2).

**Table 2:** Multivariable Cox regression analysis with HR for incident UNE in relation to BMI (kg/m^2^) subcategories as, firstly, a continuous variable and secondly stratified in participants with overweight and obesity with participants with normal weight as reference group.

	Model 1^a^	*P*- value	Model 2^b^	*P*-value
	HR (95% CI)^c^		HR (95% CI)^c^	
BMI continuous (kg/m^2^)^c^	1.06 (1.02–1.10)	<0.01	1.07 (1.03–1.11)	<0.001
Normal weight	Reference	–	Reference	–
Overweight	1.49 (1.08–2.06)	0.02	1.55 (1.12–2.15)	<0.01
Obesity	2.07 (1.31–3.28)	<0.01	2.23 (1.39–3.57)	0.001

*BMI* body mass index.

^a^Adjusted for sex and age.

^b^Adjusted for sex, age, smoking, manual work, and hypertension.

^c^HR expressed as per one unit (kg/m^2^) increase of BMI.

### Interaction and sensitivity analysis

Excluding individuals with BMI < 20 kg/m^2^ did not change the results significantly, and both overweight and obesity were still associated with incident UNE in the Cox regression model (data not shown). Likewise, excluding all participants with a follow-up time >20 years did not significantly change the results in the Cox regression models. Finally, an interaction model between BMI and smoking was created, but no significant interaction (*p* for interaction *p* = 0.40) was found.

## Discussion

The main finding from this large observational and population-based study is that high BMI is independently associated with the development of UNE in a middle-aged population without diabetes from northern Europe during >25 years of follow-up. There was an increased risk for the development of UNE in both the groups with overweight and obesity when compared to the normal-weight group, also when adjusting for confounders, such as age, sex, hypertension, smoking habits, alcohol consumption, and manual work; factors known to be risk factors for UNE. Furthermore, an exposure-response relationship was found as one increment of BMI increased the risk of UNE by ~7%. To our knowledge, this is the first study based on individuals without DM investigating the effect of an elevated BMI on an entrapment neuropathy, such as UNE. This study adds data on risk factors of UNE in individuals without DM, as we excluded all individuals with either prevalent DM at baseline or incident DM during follow-up. Trying to isolate BMI and obesity as independent risk factors for UNE is important to initiate early lifestyle intervention, but also for the development of new therapies, as lowering BMI could be a potential treatment for UNE and other entrapment neuropathies.

Our results align with several previous observational studies, which also report an association between high BMI and UNE [20–22]. There are conflicting results in the previous literature, with some studies not reporting any association between high BMI and UNE [4, 23, 24]. These studies, however, have often been comparatively small, and since UNE is a rare disease, there may be a lack of necessary statistical power [25] to detect an actual impact of BMI on the risk of UNE. Furthermore, normoglycaemic individuals with obesity have been reported to have a high prevalence of neuropathy [26]. Thus, the present study corroborates previous studies, but also provides new large-scale, longitudinal data that adds to the evidence that high BMI indeed is associated with incident UNE. The association between adipose tissue and the related entrapment neuropathy, carpal tunnel syndrome, is known, and previous Mendelian randomisation studies [27] have suggested an independent, causal relationship between obesity and CTS. As there is no published genetic data for UNE, this study adds observational data on the relationship between adipose tissue and UNE.

### Pathophysiology

The pathophysiology behind entrapment neuropathies, such as UNE, is still not fully understood. However, there is now, based on publications over the last 50 years, strong evidence on how and why DM and hyperglycaemia affect the peripheral nerve. The pathophysiology behind this glucotoxicity has been summarised elsewhere and is outside the scope of this paper [28, 29]. Neuropathy in hyperlipidemia and obesity, on the other hand, has not been as well researched as neuropathy in the presence of hyperglycaemia, especially regarding peripheral entrapment neuropathy, such as UNE and also CTS. This is so even though obesity has been proposed to be the second most important risk factor for the development of neuropathy after DM [26]. The pathobiological mechanism for how obesity affects the peripheral nerve is multifactorial and tightly linked to other components of the metabolic syndrome, such as high blood pressure, high triglycerides, low levels of HDL cholesterol, and insulin resistance [30]. Proposed pathobiological mechanisms include injury to the neurons and their related cells, particularly the Schwann cells, from LDL-cholesterol-induced oxidative stress, possibly also affecting the endoneurial microvascular circulation [11]. Also, mitochondrial dysfunction and altered mitochondrial axonal trafficking, altering the bioenergetics of the neuron, have been proposed in cellular studies [10, 31]. Finally, inflammation and the development of a proinflammatory microenvironment due to altered lipid metabolism have also been proposed as a mechanism in developing peripheral neuropathy [11]. Taken together, a high BMI might alter both the microenvironment, blood supply, and bioenergetics of the peripheral nerve, thereby possibly lowering the threshold for entrapment in, e.g., the cubital tunnel in the elbow, based on the presence of an underlying neuropathy, making the nerve trunk susceptible to entrapment [32]. This results in symptom development among the affected individuals, explaining the increased incidence of UNE in the population with overweight and obesity. The same reasoning could be applied to explain the causal relationship between obesity and CTS [27].

### Strengths and limitations

There are several strengths to this study, the main one being the large number of individuals in the MDCS cohort, enabling the study of rarer diagnoses, such as UNE. Furthermore, the long follow-up of over 25 years and the longitudinal setting enable a correct temporality between the exposure (BMI) and the outcome (UNE), which represent strengths. Finally, the quality of the baseline registrations and the availability to adjust for confounding factors, such as manual work and smoking, are additional strengths attributable to the study. Nevertheless, there might be other confounding factors not available that might affect the results, and one must keep in mind that the observational nature of this study does not provide evidence for a causal relationship between our exposures and UNE but rather associations. For example, the study does not include any genetic influence on UNE risk, a known predisposing factor in e.g., carpal tunnel syndrome [33], and one that may affect the results. Furthermore, both BMI and other detrimental factors, such as smoking habits or working conditions, might change over time. As this was only imputed once at the study baseline, the results must be interpreted with this in mind. How, for example, weight loss or weight gain over time affects the risk of UNE would be interesting to study, especially as a possible treatment for the conditions, given novel pharmacological drugs now available, particularly for the treatment of DM, but also used for obesity. Finally, the absence of impaired glucose intolerance (IGT) data represents a limitation of the study. IGT could potentially influence the risk of UNE independently of BMI, although less probable, and its inclusion might have provided an understanding of metabolic influences on UNE. Unfortunately, data on IGT is not available in the MDCS, but future research should aim to incorporate this variable to explore this relationship further. Also, there might be individuals with incipient or undiagnosed DM at baseline. Most of these individuals will, however, be excluded since most of them will be diagnosed during the long follow-up time of 25 years.

Moreover, the study only uses diagnosis codes from a hospital-based setting, thus not accounting for individuals diagnosed by physicians or occupational therapists in primary care. This might lead to an underestimation of incidence since not all individuals will be referred to secondary care. However, using only diagnoses derived from orthopaedic surgeons or hand surgeons increases the diagnostic accuracy of the study, mainly since UNE is uncommon and might not be frequently seen by many primary care physicians. Finally, as the study is conducted in a Swedish setting with mainly participants of Scandinavian descent, the findings might not be generalisable to other population groups. Thus, the study must be repeated in a different population, and this should be kept in mind when interpreting the results.

## Conclusion

High BMI is associated with the development of the entrapment neuropathy UNE, independent of age, sex, hypertension, smoking, manual work, and alcohol consumption in individuals without diabetes, indicating that obesity and high BMI are risk factors for the development of nerve entrapment disorders irrespective of DM.

## Supplementary information

SPSS Syntax

## Data Availability

The data can be applied for by researchers by contacting the steering-committee of the Malmö Diet and Cancer study (anders.dahlin@med.lu.se) and can be accessed after approval from the committee. SPSS syntax is provided as a supplemental file.
